# Development and validation of a time-varying correction factor for QT interval assessment in drug-resistant tuberculosis patients

**DOI:** 10.1016/j.ijantimicag.2025.107460

**Published:** 2025-02-06

**Authors:** Thanakorn Vongjarudech, Anne-Gaëlle Dosne, Bart Remmerie, Kelly E. Dooley, James C.M. Brust, Gary Maartens, Graeme Meintjes, Mats O. Karlsson, Elin M. Svensson

**Affiliations:** a Department of Pharmacy, Uppsala University, Uppsala, Sweden; b Janssen R&D, Beerse, Belgium; c Division of Infectious Diseases, Vanderbilt University Medical Center, Nashville, TN, USA; d Albert Einstein College of Medicine and Montefiore Medical Center, Bronx, NY, USA; e Division of Clinical Pharmacology, Department of Medicine, University of Cape Town, Cape Town, South Africa; f Wellcome Centre for Infectious Diseases Research in Africa, Institute of Infectious Disease and Molecular Medicine, University of Cape Town, Cape Town, South Africa; g Department of Pharmacy, Radboud University Medical Center, The Netherlands

**Keywords:** Tuberculosis, Tachycardia, Heart rate, QT interval, QT prolongation, Correction factors

## Abstract

**Background:**

Tachycardia associated with active tuberculosis (TB) often diminishes when patients recover from TB. Elevated heart rate (HR) may lead to suboptimal correction, complicating the assessment of QT prolongation when using standard correction factors (CFs), such as Fridericia’s formula (QTcF). Olliaro has proposed a CF for QT interval correction in pretreatment TB patients. However, the QT-HR correlation changes as HR decreases during treatment, indicating the need for time-varying correction.

**Methods:**

We developed an HR model to capture the HR normalisation during successful treatment. Sub-sequently, a time-varying CF was constructed using the estimated HR change rate. The performance of CFs to make corrected QT (QTc) independent from HR was evaluated by linear regression analyses of QTc versus HR within defined time bins.

**Results:**

The final HR model included asymptotic change in HR attributed to time on treatment, circadian rhythm cycles, M2 (bedaquiline-metabolite) concentration, and patient covariates. The time-varying CF decreased from 0.4081 to 0.33, with a half-life of 7.74 weeks. The slope (QTc/HR vs. Time) derived from the time-varying correction was not significantly different from 0 (95% CI −0.003 to 0.002), and the intercept was not significantly different from 0 (95% CI −0.089 to 0.006), demonstrating successful QT correction from pretreatment to the end of treatment.

**Conclusion:**

The time-varying CF effectively captures the dynamic QT-HR relationship during TB treatment, reducing the risk of misdiagnosing QT prolongation or unnecessary discontinuation of treatment. By addressing underestimation and overestimation issues in QT interval assessment, this method enhances drug evaluation in clinical trials and supports improved treatment decisions for TB patients.

## 1. Introduction

QT prolongation represents an extension of the duration of the ventricular action potential, which can lead to life-threatening cardiac arrhythmia, notably Torsade de pointes [1]. The treatment of TB involves a combination of multiple drugs, and some of them, such as fluoroquinolones, clofazimine, bedaquiline (BDQ), pretomanid and delamanid (DLM) [2–8], have the potential to prolong the QT interval. Additionally, metabolites of the drugs, such as metabolite 2 (M2) of BDQ and DM-6705 of DLM, can also prolong the QT [5,9,10]. This highlights the importance of accurate QT interval assessment in TB patients, as misestimations can lead to delayed detection of arrhythmic risk or unnecessary discontinuation of effective treatments (FDA-approved regimens), potentially compromising patient outcomes [11]. Because the uncorrected QT interval shortens as HR increases, it is necessary to make the QT interval independent from HR by using a CF to determine the QTc as the slope of QTc and HR is expected to be close to 0, indicating the minimal HR influence on QT [12]. Inaccurate correction can result in significant clinical consequences. For example, a positive slope indicates overcorrection, which might exaggerate QT prolongation risk, while a negative slope indicates undercorrection, potentially overlooking arrhythmic risk. Commonly, a population-based correction approach is used, where $\text{QTc}=\text{QT}/{\text{RR}}^{\text{x}}$, with x being 0.33 for Fridericia’s correction (QTcF) [13] and 0.5 for Bazett’s correction (QTcB) [14]. While QTcB and QTcF are both commonly utilized in clinical practice, QTcF typically provides better accuracy [15,16]. In addition to these approaches, linear regression-based corrections have also been proposed, such as Framingham, QTcFra = QT +0.154 (1–RR) [17] and Van de Water, QTcVa = QT–0.087 (RR–1000) [18].

Correcting the QT interval in TB patients can be challenging as active TB often increases the HR, which typically normalizes with effective TB treatment [3,8]. QTCF has been found to suboptimally correct QT intervals when HR is elevated before starting treatment, while it performed well by the end of treatment [3]. The suboptimal correction leads to bias in determining QT prolongation, as it underestimates QT at baseline and overestimates change from baseline (ΔQT) while receiving treatment. This misestimation can complicate the evaluation of drug safety, as demonstrated by ΔQTcF often increasing over time in patients receiving treatment without any QT-prolonging drug [3,6].

Olliaro et al. and Li et al. [3,8] estimated specific CFs for TB patients before receiving treatment to be 0.4081 and 0.42, respectively. However, these constant CFs do not address the changing correlation between QT and HR over time on treatment. To address this gap, our study introduces a time-varying CF (QTcTBT) that adjusts for the dynamic QT-HR relationship throughout the treatment period. This novel approach improves accuracy in QT interval correction and ensures clinical relevance by being applicable in routine practice. Our aims were to develop a model that describes the different components leading to changes in HR over time in patients treated for active TB and to establish a time-varying correction method for QT interval measurements that accounts for gradual changes in HR during the treatment period.

## 2. Material and methods

### 2.1. Data

For HR model and CF development, we utilized data from 440 participants from two phase IIb clinical trials: the C208 study [19,20] (a 2-stage, randomized, double-blind, placebo-controlled trial in newly diagnosed multidrug-resistant TB (MDR-TB) patients receiving BDQ or placebo) and the C209 study [21] (a single-arm, open-label trial in newly diagnosed MDR-TB or treatment-experienced MDR-TB patients receiving BDQ). Due to the inconsistent availability of time-matched concentrations and ECGs, the individual BDQ and M2 concentrations were predicted at the time of electrocardiography (ECG) measurements using a published population PK model [22]. For external evaluation of the HR model, the validation datasets included two different studies: *i*) A5343 DELIBERATE, a phase 2, open-label randomized, controlled trial in 82 MDR- or rifampicin-resistant TB patients receiving BDQ, DLM, or both for 24 weeks in addition to background treatment [7] and *ii*) the subset of PROBeX [23] study, a prospective cohort study of 170 MDR TB patients in South Africa, receiving a BDQ-containing regiment for 24 weeks. Each of the studies received ethical approval from appropriate local authorities, and all patients or their representatives provided written informed consent. The trials are registered on ClinicalTrials.gov (NCT00449644 for the C208 study, NCT00910871 for the C209 study, and NCT02583048 for A5343 DELIBERATE).

### 2.2. Heart rate model

#### 2.2.1. Structural models

The structural model, aiming to describe changes in HR, consisted of two components: the time-on-treatment effect and the circadian rhythm. To capture HR normalisation as patients recovered from active TB, we assumed that HR would approach a lower asymptote over time, reflecting the gradual return to a healthy baseline state. HR changes from baseline during treatment as patients recovered from active disease were investigated using linear or asymptotic models (Eqs. (1) and 2). The within-day circadian variation of HR was modelled using a cosine function with one to two oscillations, i.e., 24 and 12 h (Eq. (3)). The sum of these components represents the composite HR.

(1) TE(t)=α×t

(2) $$TE(t)=\left(HR_{\text{recovered}}-HR_{\text{baseline}}\right)\times \left(1-e^{\frac{-ln2\times t}{t_{\text{prog}}}}\right)$$

(3) $$\text{DIUR}(\text{CTIME})=A_{l}\times cos\left(\frac{2\pi \left(\text{CTIME}-\varphi_{i}\right)}{l}\right)$$

where ${\text{HR}}_{\text{baseline}}$ and ${\text{HR}}_{\text{recovered}}$ are the HR (bpm) at baseline and when patients have recovered from the disease, respectively, α is the slope of the linear change in HR, t is the time in weeks after start of treatment, ${\text{T}}_{\text{prog}}$ is the half-life of recovery process (half-life), $A_{l}$ is the amplitude (bpm) of l hr oscillation, $\varphi_{l}$ is the acrophases (h) of l hr, and CTIME is the clock time.

#### 2.2.2. Statistical models

Inter-individual variability was assessed by assuming the random effects were symmetrically distributed with a mean of 0 and a variance of $\omega^{2}$. An exponential model (Eq. (4)) was employed to constrain individual parameters to positive values:

(4) $$p_{ij}=\theta_{i}\times e^{\eta j}$$

Where $P_{ij}$ is the individual parameter estimate i for the $j^{\text{th}}$ subject, $\theta_{i}$ is the typical value of i, and $\eta_{j}$ is the deviation of the $j^{\text{th}}$ subject from the typical value of i.

#### 2.2.3. Drug effects

The effect of the main metabolite of BDQ, M2, on HR (M2EF) was explored using an ${\text{E}}_{\text{max}}$ model (Eq. 5)

(5) $$M2EF=\frac{E_{\text{max},M2}\times \text{ConcM}2}{EC50,\text{M}2+\text{ConcM}2}$$

where Emax is the maximal HR decrease (fraction), EC50 is the concentration achieving half of Emax, and ConcM2 is the M2 concentration at each ECG timepoint.

#### 2.2.4. Covariate models

Covariate relationships were included as power models for continuous covariates or conditional effects relative to the most common category. Potential effects of demographic covariates (sex, race, age, body weight, serum calcium, serum potassium, and serum albumin) were tested on ${\text{HR}}_{\text{recovered}}$ and composite HR. Potential effects of disease covariates (time to positivity of mycobacteria growth indicator tube (TTP MGIT), average derived half-life of bacterial clearance from Week 0 to 8 [24], TB-drug resistant categories, lung cavitation, and HIV status) were tested on ${\text{HR}}_{\text{baseline}},{\text{T}}_{\text{prog}}$, and composite HR. The study effect was tested on ${\text{HR}}_{\text{baseline}}$ and composite HR as there were differences in the study design between Studies C208 and C209. The effect of comedications (clofazimine and moxifloxacin) was tested on the composite HR. The selection of covariates was conducted through stepwise covariate modelling, with forward selection (P<0.05) and backward elimination (P<0.01).

#### 2.2.5. Model evaluation

Model selection was based on the objective function value (OFV,–2·log (total likelihood of data), goodness of fit, parameter uncertainty and scientific plausibility. A reduction in OFV 3.84 for a one-parameter change was considered statistically significant (P<0.05). Parameter uncertainty was determined by the standard errors or confidence interval (CI) obtained from the Fisher information matrix. Simulation-based diagnostics, i.e. visual predictive checks (VPCs), were conducted to evaluate the models’ performance. The rate of change in HR with time-on-treatment was assessed by applying the developed model from C208 and C209 to the validation datasets to compare whether there was any significant difference in the rate of HR change between training and validation data. The final HR model was employed to estimate baseline HR, recovered HR, ${\text{T}}_{\text{prog}}$, and RUV in the validation datasets while keeping all other model parameters fixed (including the effects of available covariates in the validation datasets).

### 2.3. Time-varying QT correction factor (QTcTBT)

Olliaro PL et al. and Li H et al. [3,8] proposed similar CFs of 0.4081 and 0.42, respectively, for the TB population at pretreatment. Olliaro’s CF was chosen over Li’s because it was derived from a larger cohort of 1 686 patients rather than 830, thereby providing a broader representation of the TB patient population and potentially offering greater robustness and generalizability of the correction factor. The time-varying CF was constructed using a parameter from the HR model, estimated from the C208 and C209 studies, that describes the HR change rate. The CF development was prespecified based on the assumption that the HR change rate reflects the correlation between QT and HR changes. The time-varying CF and standard CFs were evaluated in two steps based on the combined estimation and validation datasets. First, linear regression analyses were performed to investigate the performance of CFs by determining the slope of QTc versus HR within defined time bins. A slope and ${\text{r}}^{2}$ value close to 0 indicated a successful correction. Second, a linear regression model was used to investigate the change in slope over time. The slopes (QTc/HR) at the different time bins were used as data points, weighted by their standard errors. An intercept (QTc/HR slope at time 0) and a slope (QTc versus HR) over time bins are expected to be close to 0 for successful correction.

### 2.4. Standard endpoints QTc prolongation assessment of Studies C208 and C209

Studies C208 & C209 were investigated for three standard end-points: absolute QTc, ΔQTc, and placebo-corrected change from baseline in QTc (ΔΔQTc) over the study duration (from pretreatment until week 24) due to the availability of placebo data, to compare the secular trend in the placebo arm when using QTcF or QTcTBT. Pretreatment (baseline) QTc was defined as Day −1, 5 h postdose for the 5-hour assessments, and Day −1, predose for all other assessments. The summary statistics were provided over the following time-bins: *i*) pretreatment, *ii*) Week 0 to 1, *iii*) Week 2 to 3, *iv*) Week 3 to 4, *v*) Week 5 to 8, *vi*) Week 9 to 12, *vii*) Week 13 to 20, and *viii*) Week 20 to 24.

### 2.5. Software

The model development and simulation were performed using NONMEM (ICON plc, Hanover, MD, USA) version 7.4.4 and 7.5 in a Linux operating system on the Uppsala Multidisciplinary Center for Advanced Computational Science (UPPMAX) cluster. Perl-speaks-NONMEM (PsN, version 5.3.0, [http://psn.sourceforge.net/docs.php]) was used for aiding NONMEM runs [25]. The statistical software R (version 4.2.2, The R Project for Statistical Computing, [www.r-project.org]) together with R packages, Xpose4, ggplot2, and tidyverse, were used for data management, exploratory analyses, diagnostic graphics, and post-processing of the data and NONMEM outputs.

## 3. Results

### 3.1. Patients and data

A total of 440 patients from the studies C208 and C209, which contributed to 18,657 HR observations and 18 495 QT observations, were included in the HR model and CF development. Individual ECG measurements were used; triplicate ECGs from the same time point were not averaged, or absolute single ECGs (see supplementary document S1). ECG measurements earlier than the Day −1 visit and after the end of treatment were excluded. A summary of patients’ demographic and baseline characteristics is shown in Table 1.

### 3.2. HR model

The final HR model comprises baseline HR, an asymptotic time-on-treatment effect, 24-hour and 12-hour circadian rhythms, and an effect of M2 concentrations, as described in Supplementary S2, and the code of the final HR model is presented in Supplementary S3. Patient-related RUV characterised the residual unexplained variability (RUV) and between-triplicates RUV, allowing for the handling of residual error magnitude for both patients and triplicates. The final parameter estimates and uncertainty from the final model are shown in Table 2.

The typical baseline HR, reflecting that of untreated patients with active TB, was estimated at 78.2 bpm. After receiving TB treatment, patients’ HR recovered to 73.1 bpm, with a half-life $\left({\text{T}}_{\text{prog}}\right)$ of 7.74 weeks (95% CI: 5.17–10.27 weeks). The 24, 12-hour cycle of the circadian rhythm indicated that the HR reached its maximum approximately at 2.25 pm, with a maximum amplitude of 4.8 bpm.

The inhibitory effect of M2 on HR was mild, with an estimated Emax of 17.9% and an EC50 of 2,600 ng/mL. For M2 concentrations of 300 ng/mL (close to the observed mean at week 2), HR is estimated to decrease by 2% (95% CI: 1%–3%) compared to its value without BDQ treatment, equivalent to a 1.6 bpm reduction from a 78.2 bpm baseline. The estimated inter-individual variability of EC50 was found to be high, indicating significant variability in the magnitude of the effect of M2 within the population.

An alternative assumption, the parameter estimates of the scenario where no M2 effect is involved in HR, are summarised in Supplementary S4. The estimated ${\text{T}}_{\text{prog}}$ from the model without the M2 effect was similar to the estimate from the final model (5.88 weeks vs. 7.74 weeks, respectively), as the difference fell within the 95% CI for ${\text{T}}_{\text{prog}}$ in the final model.

**Fig. 1.**
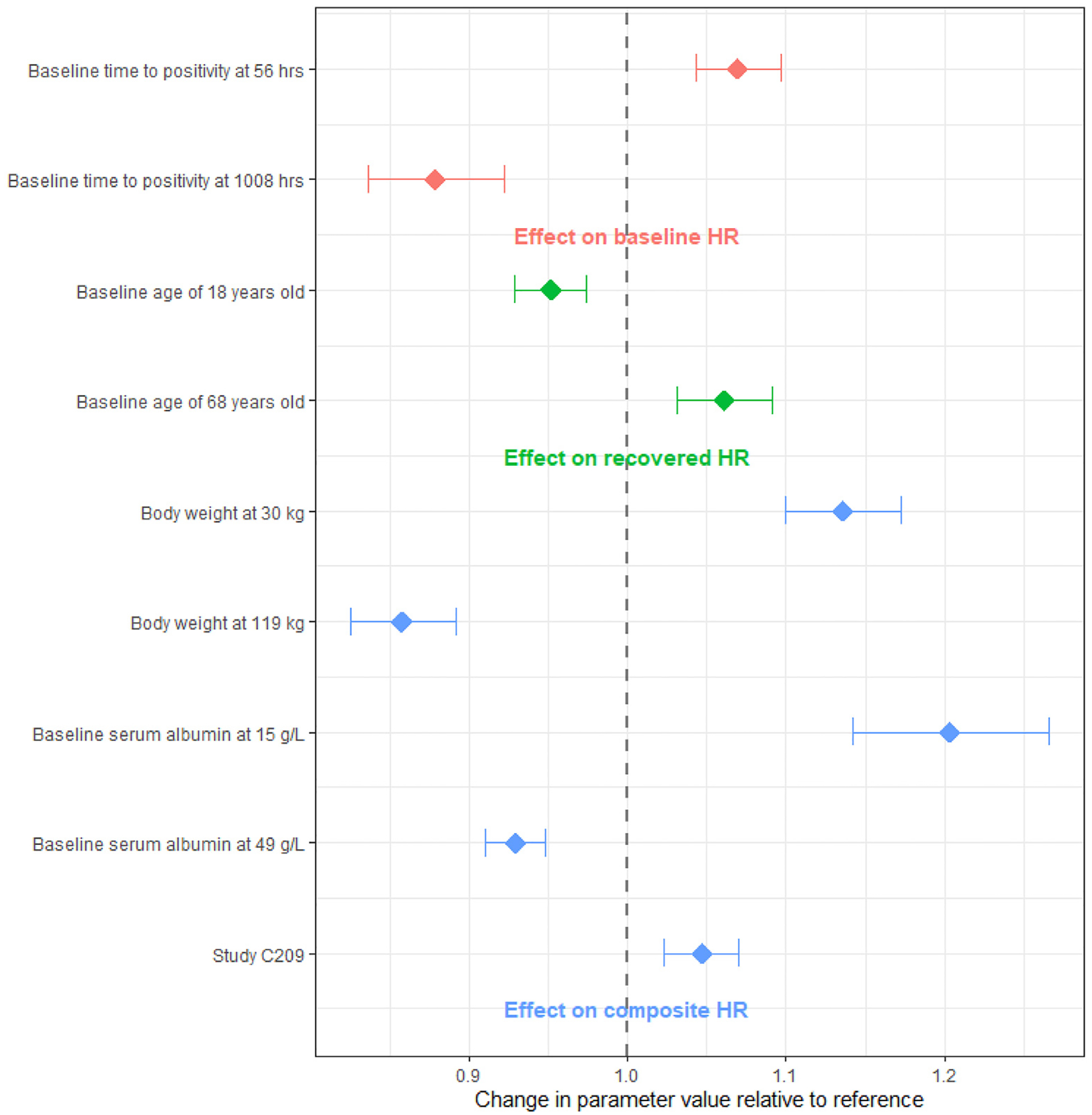
Forest Plot of Covariate Effects from the Final HR model. *H* = hour; HR = heart rate; MGIT = mycobacteria growth indicator tube. The vertical dashed line represents the reference values for continuous covariates, including baseline time to positivity, age, time to positivity, and serum albumin; the observed maximum and minimum values are displayed. For categorical covariates, the comparison is between Study C209 and Study C208. The bands depict the 90% confidence interval around the point estimate, showing the change in baseline, recovered, or composite HR from the reference maximum and minimum observed values (subject albumin of 35 g/L for baseline HR, subject of 33 years of age for recovered HR, and subject in Study C208 of 33 years of age, with body weight of 56 kg, albumin of 35 g/L MGIT of 230.5 h for composite HR).

The covariate effects for the final HR model were modelled as described in Supplementary S2 and are illustrated in Fig. 1. Patients’ age, body weight, baseline serum albumin, and bacterial burden at the start of treatment (quantified by the TTP MGIT) were statistically significant. A study effect was included due to the observed typically lower HR in the C209 population compared to C208.

Parameter estimates for the validation datasets are presented in Supplementary S5. The covariate model from the final model was adapted to the validation datasets based on data availability. The effects of the study and the impact of TTP MGIT were not applied to A5343 data, and no covariate model was applied to PROBeX. The baseline heart rate (HR) of A5343 and PROBeX was estimated to be higher than that of C208&C209, which were 83 and 85.4 bpm, respectively. Additionally, the estimated HR at recovery from A5343 was 63.3 bpm, which was lower than that from C208&C209, while the estimated HR at recovery from PROBeX was similar to that from C208&C209, which was 73.1 bpm. The estimated ${\text{T}}_{\text{prog}}$ values for A5343 and PROBeX were 8.51 weeks and 9.46 weeks, respectively, falling within the ${\text{T}}_{\text{prog}}$ estimated from the C208 and C209 datasets.

**Fig. 2.**
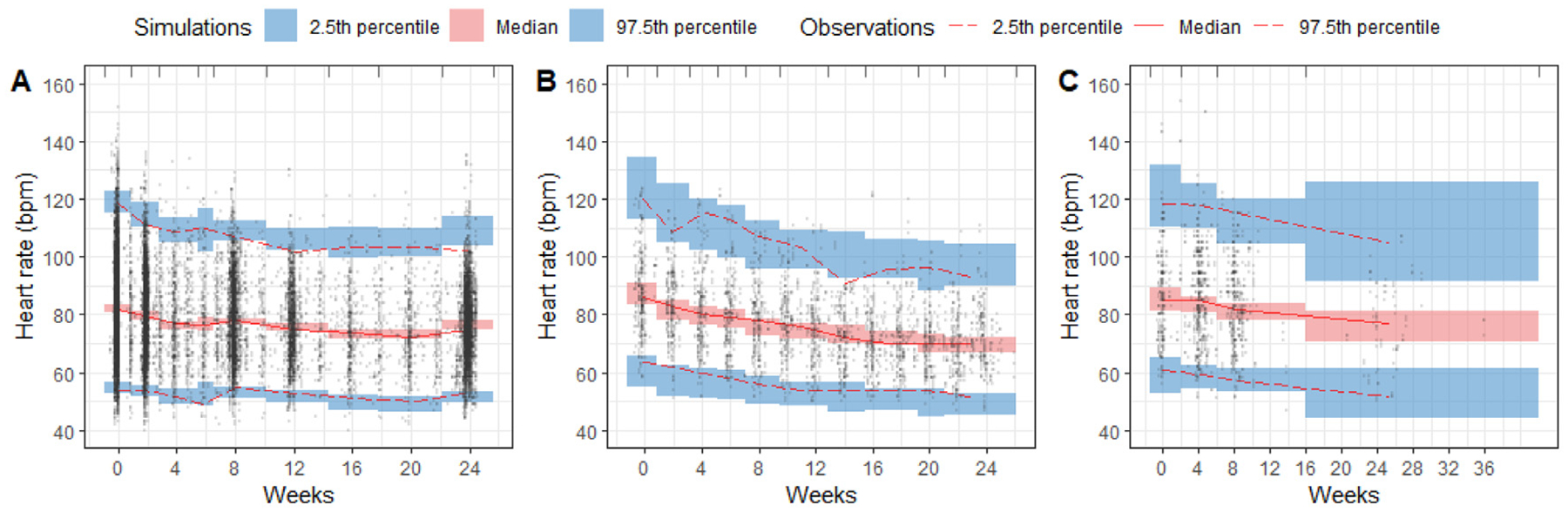
Visual predictive check of heart rate over time after the start of treatment of final models: (A) C208 and C209 development dataset; (B) A5343 validation dataset; (C) PROBeX validation dataset. Black dots are the observed HR measurement. Solid lines represent observed data for the median, while dashed lines represent observed data for the 2.5th and 97.5th percentiles. Shaded areas show 95% CI for the 2.5th median and 97.5th percentiles.

The visual predictive checks (VPCs) of the final model on development and validation datasets are shown in Fig. 2. The final model adequately captured the observations for all datasets.

### 3.3. Time-varying correction factor development

**Fig. 3.**
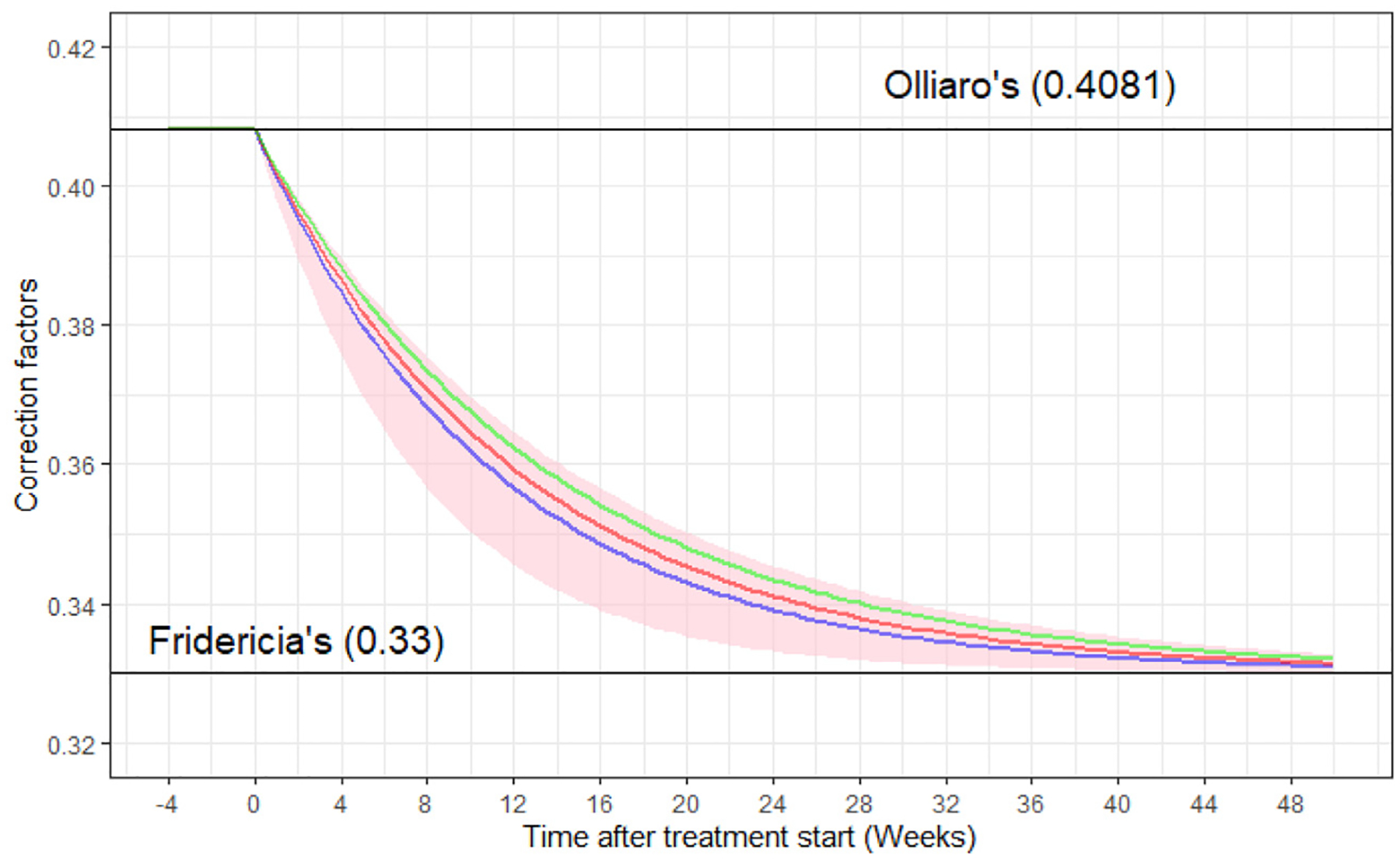
Time-varying QT Correction Factor Over Time where the blue line represents the calculated QT correction factors from estimated typical value of ${\text{T}}_{\text{prog}}$ from C208 and C209 studies (7.74 weeks) with the pink shade calculated changed in QT correction factor from represents the lower bound and upper bound of the 95% confidence interval (CI) for ${\text{T}}_{\text{prog}}$ (5.17–10.27 weeks). The red and green solid line represents the calculated QT correction factor from estimated ${\text{T}}_{\text{prog}}$ from A5343 and PROBeX, which were 8.51 and 9.46 weeks, respectively.

In preliminary analyses, linear regression-based formulas, i.e. QTcFra and QTcVa, were assessed but demonstrated inferior performance compared to the log-linear methods. For instance, OFVs for QTcF and QTcB were 113 034 and 115 079, respectively, whereas QTcFra and QTcVa produced higher OFVs of 152 116 and 145 137, indicating poorer performance. Consequently, we selected the log-linear formula for further CF development. Based on data from Studies C208 and C209, we observed that QTcB consistently over-corrected the QT interval throughout the treatment period. QTcF successfully made QTc independent from HR at the end of treatment but undercorrected before and during early treatment. In contrast, the CF proposed by Olliaro et al. (QTcO) initially performed well during early treatment but overcorrected at later time points (supplementary S6). Given the knowledge that the change in HR drives the correlation between HR and QT, it was assumed that the rate at which HR changes could inform the rate of change from QTcO (0.4081) to QTcF (0.33) in the time-varying CF. The estimated ${\text{T}}_{\text{prog}}$ parameter from the asymptotic function in the final HR model (7.74 weeks) was utilised to describe the change from 0.4081 to 0.33, described in Equation 6 and implemented as ${\text{QT}}_{\text{uncorrected}}/{\text{RR}}^{\text{CF}(\text{t})}$ to provide the new QT correction for TB population, QTcTBT. The change in CF over time is illustrated for the point estimate of ${\text{T}}_{\text{prog}}$ and the bounds of its 95% CI in Fig. 3.

(6) $$CF(t)=0.4081-0.0781\times \left(1-e^{\frac{-ln2\times t}{7.74}}\right)$$

Where CF(t) is the correction factor as a function of time, t is time in weeks after the start of TB treatment.

### 3.4. Time-varying QT correction factor evaluation and validation

**Fig. 4.**
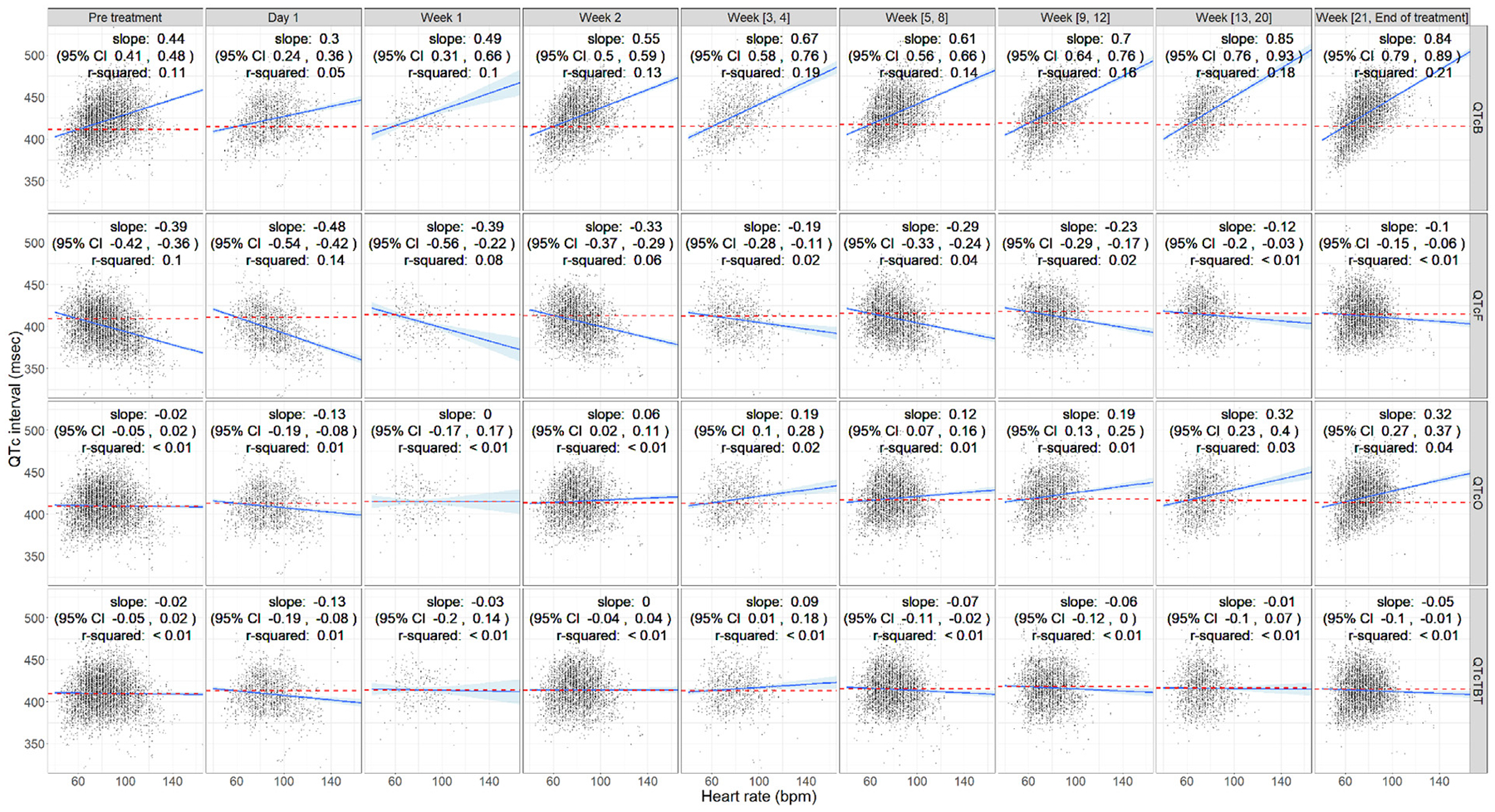
Relationship of QTc vs HR Stratified by Time Visit for Different Correction Methods (QTcB, QTcF, QTcO, and QTcTBT) from the pooled development and validation datasets. The linear regression line is blue, with the 95% confidence interval represented by the light blue shaded area. The red dashed line indicates the QTc at a heart rate of 60 bpm. QTcB: QT correction with Bazett’s formula (0.5), QTcF: QT correction with Fridericia’s formula (0.33), QTcO: QT correction with Olliaro’s formula (0.4081), QTcTBT QT correction with a time-varying correction formula, the end of treatment: week 24.

The results of the linear regression analyses evaluating the adequacy of QTcTBT alongside other correction factors (QTcB, QTcF, and QTcO) are presented for the pooled development and validation datasets in Fig. 4. QTcTBT demonstrated superior performance as a time-varying correction factor, effectively maintaining QT independence from HR throughout the entire treatment period. The relationship between QTc and HR in each dataset can be found in Supplementary S6. Additionally, the off-treatment (placebo) QTc analysis from the C208 trial is presented in Supplementary Fig. S7.

QTcTBT demonstrated slopes close to zero throughout treatment, indicating stable and reliable correction of QT intervals from the start through the end of therapy. In contrast, QTcB consistently overcorrected, and although QTcF initially undercorrected, the slope approached zero, −0.1 (95% CI −0.15, −0.06), at the end of treatment. QTcO successfully corrected QT during the pretreatment period with a slope of −0.02 (95% CI −0.05, 0.02), but it over-corrected from Week 2 onward.

To further quantify these differences, a linear regression model assessing slopes over time-bins showed that the intercept for QTcTBT was not significantly different from 0, indicating successful correction at the start of treatment. Its slope (QTc vs HR) over time was −0.001 (95% CI −0.003, 0.002), suggesting no drift in correction quality. In comparison, the intercept for QTcB and QTcF was estimated at 0.459 (95% CI 0.399, 0.519) and −0.383 (95% CI −0.419, −0.348), respectively, indicating overcorrection and under-correction at the start of treatment, respectively. Conversely, the intercepts for QTcO and QTcTBT were not significantly different from 0, suggesting successful QTc correction at the start of treatment. The slope (QTc versus HR) over time-bins for QTcB, QTcF, and QTcO exhibited increasing slopes over time (0.018, 0.012, and 0.015, respectively), indicating an inconsistency in correction performance.

The QTc vs HR analysis for off-treatment further supports this, as QTcTBT outperformed QTcF by aligning more time-bins (8 vs.3) with the expected QTc line at a heart rate of 60 bpm (Supplementary S7).

The sensitivity analysis of QTcTBT is shown in Supplementary S. Ranges within a CI from 5.17 to 10.27 weeks around the typical ${\text{T}}_{\text{prog}}$ of 7.74 weeks were determined by the number of time bins where the slope did not significantly differ from 0. The number of time-bins of QTcTBT calculated from Tprog values of 5.17, 10.27, and 7.74 weeks were similar, except for one additional time bin (Week 9–12) when using the lower bound of 5.17 weeks. This supports the robustness of QTcTBT’s ability to make QT independent from HR. Additionally, the ${\text{T}}_{\text{prog}}$ estimated from A5343 and PROBeX were within this range, supporting the adequacy of using 7.74 weeks as the representative rate of change in HR in general.

### 3.5. Standard endpoints QTc prolongation assessment of Studies C208 and C209

**Fig. 5.**
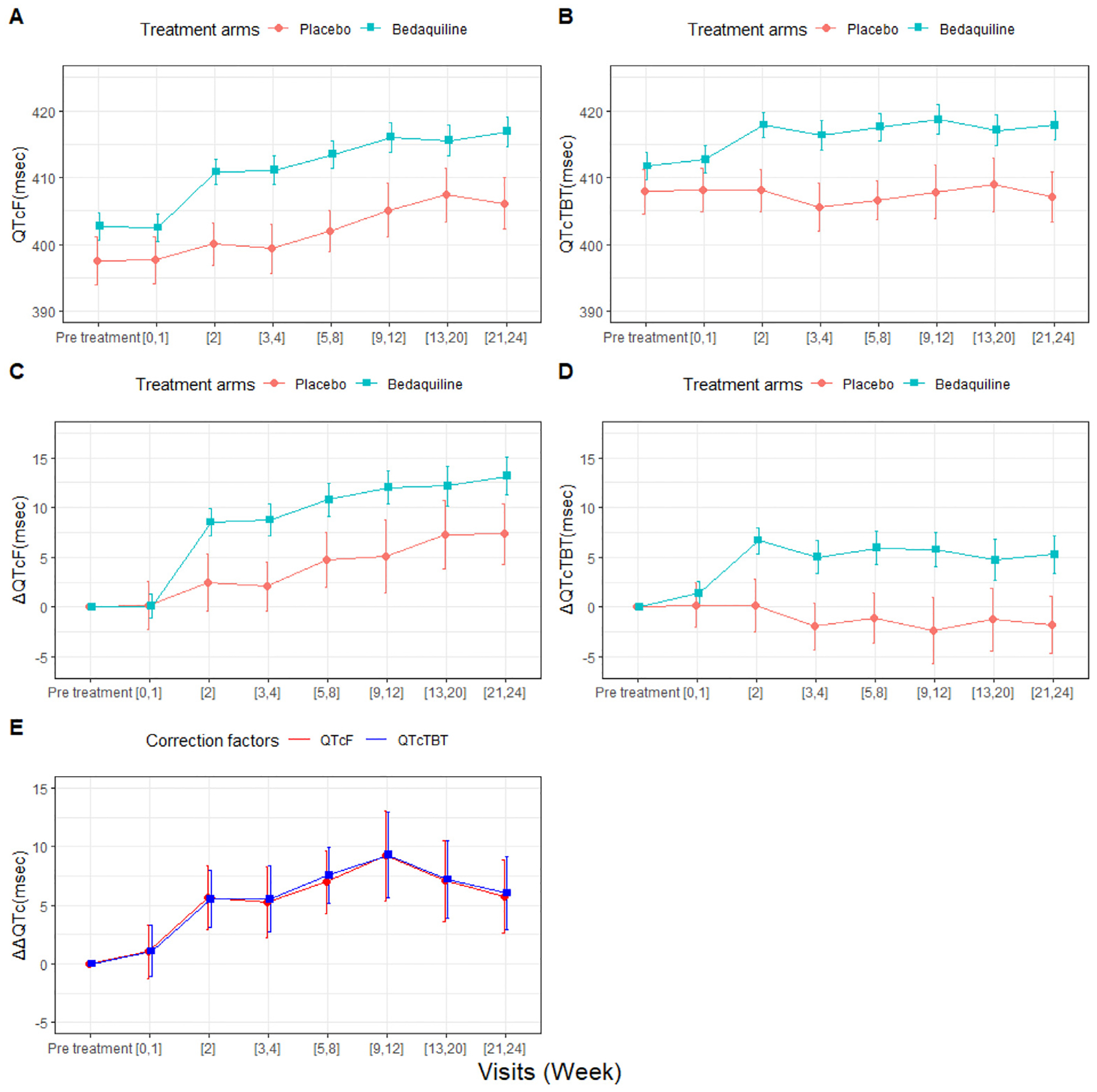
The mean and 95% confidence interval of the standard endpoints over time-bins of absolute QTcF (A) and QTcTBT (B), the change in QTc from baseline (ΔQTc) for QTcF (C) and QTcTBT (D), and the placebo-corrected changes from baseline in QTc (ΔΔQTc) for QTcF and QTcTBT from the C208 study (E).

Absolute QTc and ΔQTc showed an increasing trend over time in the placebo arm when using QTcF, but QTcTBT remained stable, as shown in Fig. 5. No difference was observed in ΔΔQTcF and ΔΔQTcTBT. The summary statistics at different time intervals can be found in Supplementary S9.

## 4. Discussion

We developed an HR model that accurately described HR normalisation over time in TB patients receiving effective treatment. Despite differences in treatment regimens and patient populations, similar HR change rates were observed across both development and validation datasets. This consistency suggests that our model’s assumptions are robust and that the time-on-treatment effect on HR is generalisable across various clinical scenarios.

The HR change rate was utilised to construct a time-varying CF that starts from an empirically derived pretreatment CF (QTcO) and transitions towards a standard CF (QTcF) by the end of treatment. QTCO performed well in the pretreatment period because it was developed based on the pretreatment group that the elevated HR state observed, and QTcF showed good performance at the end of the treatment when HR had normalised. By leveraging the estimated HR change rate, the time-varying CF (QTcTBT) aligns with QTcO at baseline and smoothly adjusts toward QTcF as treatment progresses, ensuring appropriate correction throughout. This implies that QTCO is preferable for patients prior to TB treatment, while QTcF becomes appropriate for those who have recovered or healthy individuals.

The performance of the time-varying CF for the TB population (TBT) remained robust regardless of the chosen rate of change. Sensitivity analyses showed that varying ${\text{T}}_{\text{prog}}$ within its 95% CI did not significantly affect correction accuracy (Supplement S7), and QTcTBT maintained slopes and intercepts close to zero for QTc/HR over time. Its robustness is further supported by the slope and intercept of QTc/HR versus time for QTcTBT, which did not differ from 0, indicating successful correction at any time point during treatment. In contrast, the slopes of other CFs significantly demonstrated changes in QTc/HR throughout treatment. Additionally, the result showed that QTcF can be reliably used to calculate QTc after week 24. The zero correlation may not be able to prove the accuracy of the developed CF, especially when the drug affects both QTc interval and HR [26,27]. Although M2’s impact on HR was minimal in this case, we examined the QTc and HR correlation within the placebo arm and considered standard endpoints to evaluate our approach further. In the placebo arm, the QTcTBT outperformed other CFs, and the performance of QTcTBT is supported by the standard endpoint of the QTc assessment. QTcTBT remained stable, and QTcF showed an increasing trend in the placebo arm over time, underscoring the time-varying approach’s clinical relevance in avoiding inflated QT prolongation estimates. These findings highlight QTcTBT’s adaptability and potential generalizability within the TB population while acknowledging that faster or slower HR recovery rates in future treatment scenarios may necessitate re-estimating ${\text{T}}_{\text{prog}}$.

A correction method that does not account for varying HR biases assessments such as absolute QTc and ΔQTc unless data from an adequate control treatment are available to estimate the difference in ΔQc versus placebo. Our results demonstrated that using QTCF at pretreatment or early in treatment may underestimate QTc intervals at elevated HRs, potentially leading to inappropriate initiation of QT-prolonging drug. On the other hand, patients may be contraindicated due to QTc > 500 ms resulting from the misdetection of QT prolongation before and early during treatment when using QTcF. Conversely, overestimating ΔQTcF later in treatment might result in false detection of QT prolongation, potentially leading to unnecessary treatment disruption and limiting access to an effective treatment regimen [11,23]. Although a placebo correction (ΔΔ) is able to remove the bias from CF, it is limited to drug development settings with a placebo arm available. Moreover, it is impractical for most clinical studies or practice, especially with TB patients on complex treatment regimens.

While the individual correction has been proven to be more accurate than the population approach [28], it requires a significant number of data points within an individual to derive the subject-specific CF. The decrease in HR during treatment may reflect changes in an individual’s physiology, and the optimal individual CF should change over time, making it practically infeasible in clinical routine. Previously, the model-based concentration-QT analyses, where the time on treatment is explicitly modelled, were proposed as an alternative [6]. However, this method requires highly skilled personnel, making them less suitable for clinical practice. In contrast, QTcTBT is straightforward to apply, as illustrated in Supplement S10 and via the online calculator (https://thanakornv.github.io/qtctbt/), and can, therefore, aid both in drug development and in routine clinical settings.

This novel correction method may have limitations that QTcTBT assumes a typical rate of HR recovery, which may not hold for patients who do not respond to treatment as expected. In such cases, its accuracy may diminish similarly to QTcF or QTcB. Another potential limitation is that while ${\text{T}}_{\text{prog}}$ demonstrated effectiveness in four studies, its applicability to all future treatment regimens or patient populations may vary. Further validation in diverse cohorts and treatment scenarios would strengthen the utility of QTcTBT and ensure its broader clinical applicability.

## 5. Conclusion

The novel time-varying CF significantly improves the accuracy of QT interval assessment for TB patients undergoing QT-prolonging treatments by adapting to HR changes over time. By minimising bias seen with constant correction methods and providing a more reliable assessment of QT prolongation, it enhances the interpretation of drug effects in clinical trials and supports safer, more informed treatment decisions for patients with TB receiving QT-prolonging drugs. While the current findings demonstrate its effectiveness across various treatment regimens, further validation in diverse clinical populations and under different treatment protocols will strengthen its utility and applicability in broader clinical practice.

## Supplementary Material

Supplementary Material

## Figures and Tables

**Table 1:** Demographic and baseline characteristics of the patients included in the development and validation datasets of the HR model.

	Development dataset				Validation datasets	
	Study C208 Placebo	Study C208 BDQ	Study C209 BDQ	Total C208&C209 (Used for development)	A5343	PROBeX
N	105	102	233	440	84	170
Sex, n (%)						
Male	66 (62.9%)	70 (68.6%)	150 (64.4%)	286 (65%)	63 (75%)	79 (46%)
Age (years)						
Median	34	32	32	33	34	33
Range	18–61	18–63	18–68	18–68	18–73	28–41
Body weight at baseline (kg)						
Median	53	54	57	55	52	56
Range	35–83	37–81	30–113	30–113	34–83	49–63
Race, n (%)						
White	13 (12.4%)	8 (7.8%)	60 (25.8%)	81 (18.4%)	1 (1%)	2 (1%)
Black	40 (38.1%)	42 (41.2%)	75 (32.2%)	157 (35.7%)	38 (45%)	140 (82%)
Hispanic or Latino	15 (14.3%)	13 (12.7%)	0 (0%)	28 (6.4%)	0 (0%)	0 (0%)
Asian	6 (5.7%)	9 (8.8%)	90 (38.6%)	105 (23.9%)	0 (0%)	0 (0%)
Other	31 (29.5%)	30 (29.4%)	8 (3.4%)	69 (15.7%)	45 (54%)	28 (16)
TB-drug resistance categories, n (%)						
TB drug sensitive	4 (3.8%)	3 (2.9%)	3 (1.3%)	10 (2.3%)	6 (7.1%)	0 (0%)
MDR TB	63 (60%)	70 (68.6%)	90 (38.6%)	223 (50.7%)	62 (73.8%)	0 (0%)
Pre-XDR TB	16 (15.2%)	17 (16.7%)	43 (18.5%)	76 (17.3%)	0 (0%)	0 (0%)
XDR TB	5 (4.8%)	3 (2.9%)	37 (15.9%)	45 (10.2%)	0 (0%)	0 (0%)
Not available	17 (16.2%)	9 (8.8%)	60 (25.8%)	86 (19.5%)	16 (19%)	170 (100%)
HIV status, n (%)						
HIV positive	19 (18.1%)	11 (10.8%)	11 (4.7%)	41 (9.3%)	30 (35.7%)	105(62)
Not available	0 (0%)	6 (5.9%)	11 (4.7%)	17 (3.9%)	3 (3.6%)	0 (0%)
QT-prolonging comedication, n (%)						
Clofazimine	0 (0%)	0 (0%)	25 (10.7%)	25 (5.7%)	NA	167 (98.2%)
Moxifloxacin	4 (3.8%)	3 (2.9%)	2 (0.9%)	9 (2%)		44 (25.9%)
Serum albumin at baseline (mg/dl)						
Median	31	34	38	35	34	34
Range	17–46	15–49	21–49	15–49	21–44	29–39
Serum calcium at baseline (IU/L)						
Median	2.53	2.54	2.43	2.48	3.3	NA
Range	2.28–2.84	2.3–2.82	2.15–2.86	2.15–2.86	1.16–4.4	
Serum potassium at baseline (IU/L)						
Median	4.3	4.4	4.1	4.3	4.2	NA
Range	3.4–5.8	3.6–.8	2.7–5.4	2.7–5.8	3–5.5	
TTP MGIT at baseline (h)						
Median	160.5	169.67	346	230.5	NA	NA
Range	56–1008	75.33–1008	78.67–1008	56–1008		
Lung cavitation at baseline, n (%)						
Positive	77 (73.3%)	80 (78.4%)	148 (63.5%)	305 (69.3%)	NA	NA

BDQ = bedaquiline, TTP = time to positivity, MGIT = mycobacteria growth indicator tube, N = number of participants, NA = not available.

**Table 2:** Parameter estimates of the final HR model.

Description		Parameter estimate (%RSE) [95%CI]	Inter individual variability %CV (%RSE)
Time on treatment	Baseline HR (bpm)	78.2 (1.2) [76.3, 80.1]	15 (4)
	Recovered HR (bpm)	73.1 (1.4) [71.1, 75.2]	15.4 (6)
	${\text{T}}_{\text{prog}}$ (weeks)	7.74 (16.8) [5.17, 10.27]	
Circadian rhythm	Amplitude 24 h (bpm)	6.2 (13.6) [4.5, 7.9]	95.3 (9)
	Peak time 24 h (clock time)	15.7 (1.3) [15.3, 16.2]	
	Amplitude 12 h (bpm)	1.65 (19.7) [1, 2.3]	95.3 (9)
	Peak time 12 h (clock time)	10.1 (4.8) [9.2, 11.1]	
	Box-Cox shape for IIV amplitudes	−0.77 (20.9) [−1.09, −0.46]	
M2 effect on HR	${\text{E}}_{\text{max}}$ (fraction)	0.179 (11.1) [0.14,0.218]	
	${\text{E}}_{50}$ (ng/mL)	2600 (13) [1936, 3260]	883.4 (2)
Covariate effects	Effect of study on HR (Study C209 vs C208)	0.047 (31.3) [0.018, 0.076]	
	Effect of time varying body weight on HR	−0.2 (15.2) [−0.26, −0.14]	
	Effect of baseline serum albumin on HR	−0.22 (17.6) [−0.3, −0.14]	
	Effect of baseline TTP MGIT on baseline HR	−0.05 (22.9) [−0.07, −0.03]	
	Effect of age on recovered HR	0.08 (30.1) [0.03, 0.13]	
Residual error model	Proportional RUV (%)	8.4 (2)	23.6 (8)
	Additive replicated-specific RUV (bpm)	2.7 (1.9)	38.5 (4)

bpm = beats per minutes, CI = confidence interval, CV = coefficient of variation EC_50_ = half maximum effect concentration, E_max_ = maximum effect, h = hour, HR = heart rate, IIV = inter individual variability, TTP = time to positivity, MGIT = mycobacteria growth indicator tube, RSE = residual standard error; RUV = residual unexplained variability, T_prog_ = time to reach 50% of recovered HR (half-life), $\%\text{CV}=\sqrt{e^{\omega^{2}}-1}$, Correlation between IIV: Baseline HR: recovered HR = 0.41 (%RSE 9), Baseline HR: amplitude = −0.26 (%RSE 13.8), Recovered HR: amplitude = −0.421 (%RSE 10.2), Proportional RUV: additive triplicate-specific RUV = 0.38 (%RSE 12).
