# Supplementary material for: Development and validation of a time-varying correction factor for QT interval assessment in drug-resistant tuberculosis patients
Source: Int J Antimicrob Agents. Author manuscript; Available in PMC 2025 May 27. (PMC12107617; doi:10.1016/j.ijantimicag.2025.107460)
Supplement: Supplementary Material [file NIHMS2080384-supplement-Supplementary_Material.docx]

**Supplementary data**

**Development and Validation of a Time-Varying Correction Factor for QT Interval Assessment in Drug-Resistant Tuberculosis Patients**

Thanakorn Vongjarudech^1^, Anne-Gaëlle Dosne^2^, Bart Remmerie^2^, Kelly E Dooley^3^, James C M Brust^4^, Gary Maartens^5^, Graeme Meintjes^5,6^, Mats O Karlsson^1^, Elin M Svensson^1,7^

^1^Department of Pharmacy, Uppsala University, Sweden, ^2^Janssen R&D, Beerse, Belgium, ^3^Division of Infectious Diseases, Vanderbilt University Medical Center, Nashville, Tennessee, USA, ^4^Albert Einstein College of Medicine and Montefiore Medical Center, Bronx, NY, USA, ^5^Division of Clinical Pharmacology, Department of Medicine, University of Cape Town, Cape Town, South Africa, ^6^Wellcome Centre for Infectious Diseases Research in Africa, Institute of Infectious Disease and Molecular Medicine, University of Cape Town, Cape Town, South Africa, ^7^Department of Pharmacy, Radboud university medical center, The Netherlands

**Table of contents Page**

Supplementary 1 (S1) : ECG sampling times in study C208 and C209 2

Supplementary 2 (S2): The final heart rate (HR) model and Covariate effects 3

Supplementary 3 (S3): The final model NONMEM code 4

Supplementary 4 (S4): Parameter estimates from the final HR model without M2 Effect 7

Supplementary 5 (S5): Parameter estimates of the final HR model for validation datasets 8

Supplementary 6 (S6): Relationship of QTc vs HR Stratified by Time Visit 9

Supplementary 7 (S7): Relationship of QTc vs HR Stratified by Time Visit for
off-treatment (placebo) from C208 datasets 12

Supplementary 8 (S8): Relationship of QTc vs HR Stratified by Time visit for a time
varying correction method calculated with the typical, upper and lower boundary of 95%CI
of T_prog_ from the pooled development and validation datasets 13

Supplementary 9 (S9): Summary Statistics for absolute change from baseline(Δ),
placebo-corrected change from baseline (ΔΔ) of QTcF and QTcTBT at Different
Time Intervals 14

Supplementary 10 (S10): Example of QTcTBT calculation 15

**Supplementary 1 (S1): Electrocardiogram (ECG) sampling times in studies C208 and C209**

| **ECGs Sampling times^a^** | **Studies^b^** | | | |
| --- | --- | --- | --- | --- |
|  | **C208** | **C209** | **A5343** | **PROBeX** |
| Screening^c^ | Triplicate | Single | Triplicate | Triplicate |
| Day -1^d^ | Triplicate | Triplicate | Triplicate | - |
| Day 1 | Triplicate | Triplicate | - | - |
| Week 1 | Single | - | - | - |
| Week 2 | Triplicate | Triplicate | Triplicate | - |
| Week 3 | Single | - | - | - |
| Week 4 | Single | Single | Triplicate | Triplicate |
| Week 5 | Single | - | - | - |
| Week 6 | Single | - | Triplicate | - |
| Week 7 | Single | - | - | - |
| Week 8 | Triplicate | Triplicate | Triplicate | Triplicate |
| Week 10 | Single | - | Triplicate | - |
| Week 12 | Single | Triplicate | Triplicate | - |
| Week 14 | Single | - | Triplicate | - |
| Week 16 | Single | Single | Triplicate | - |
| Week 18 | Single | - | Triplicate | - |
| Week 20 | Single | Single | Triplicate | - |
| Week 22 | Single | - | Triplicate | - |
| Week 24 | Triplicate | Triplicate | Triplicate | Triplicate |

^a^ For C208 and C209, A single ECG was performed up to 1 hour before drug intake (8 AM), and triplicate ECGs were performed just before drug intake (8 AM) and 5 hours post-dose. At weeks 8 (for stage 1) and 24 (for stage 2) in the C208 study, additional triplicate ECGs were performed at 36 hours and 48 hours post-dose. The ECG was measured supine after at least 5 minutes of rest.

^b^ C208, C209, and A5343 were clinical trial studies, while PROBeX was a prospective observational cohort study.

^c^ For C208 and C209, the screening visit was performed 1 week prior to Day 1. For A5343, the screening visit was performed within 72 hours prior to entry.

^d^ In the A5343 study, ECGs were done on day -1 or day 0.

**Supplementary 2 (S2): The final heart rate (HR) model and Covariate effects**

The time-on-treatment effect (TE), which describes the changes in HR from baseline to the recovered state during treatment, was modelled using an asymptotic model (Equation A).

$TE\left( t \right)=\left( {HR}_{recovered}- {HR}_{baseline} \right)\times(1-e^{\frac{-ln2\times t}{t_{prog}}})$ (Eq. A)

Where HR_baseline_ and HR_recovered_ are the HR (bpm) at baseline and when patients have recovered from the disease, respectively, *t* is the time in weeks after the start of treatment, T_prog_ is the half-life of the recovery process

The within-day circadian variation of HR (DIUR) was modelled using a cosine function with 24-hour and 12-hour oscillations (Equation B).

$DIUR(CTIME)=A_{24}\times\cos\left( \frac{2\pi(CTIME-\varphi_{24})}{24} \right)$ + $A_{12}\times\cos\left( \frac{2\pi(CTIME-\varphi_{12})}{12} \right)$ (Eq. B)

Where *A_l_* is the amplitude (bpm) of *l* hr oscillation, *φ_l_* is the acrophases (h) of *l* hr, and CTIME is the clock time.

The effect of M2 (M2EF) on HR was modelled using an Emax model (Equation C).

$M2EF= \frac{E_{max,M2}\cdot ConcM2}{EC50,M2+ConcM2}$ (Eq. C)

Where Emax is the maximal HR decrease (fraction), EC50 is the concentration achieving half of Emax, and Conc is the individual model predicted M2 concentration at each ECG timepoint.

The final HR model was constructed as a composite of baseline HR, an asymptotic time-on-treatment effect, 24-hour and 12-hour circadian rhythms, and the effect of M2 plasma concentrations (Equation D).

$HR=\left( {HR}_{baseline}+\left( {HR}_{recovered}- {HR}_{baseline} \right)\times\left( 1-e^{\frac{-ln2\times t}{t_{prog}}} \right) +DIUR \right)\times\left( 1-M2EF \right)$
 (Eq. D)

The full covariate effects were implemented to each of component of the final HR model as follows (Equation E, F, G)

$HR=HR\times\cdot(1+\theta_{StudyC209}\cdot{Study}_{C209,k})\times{(\frac{{TBW}_{k}}{{TBW}_{median}})}^{\theta_{twt}}\times{(\frac{{BALB}_{k}}{{BALB}_{median}})}^{\theta_{BALB}}$(Eq. E)

${HR}_{baseline}={HR}_{baseline}\times{(\frac{{BMGIT}_{k}}{{BMGIT}_{median}})}^{\theta_{BMGIT}}$ (Eq. F)

${HR}_{recovered}={HR}_{recovered}\times{(\frac{{Age}_{k}}{{Age}_{median}})}^{\theta_{Age}}$ (Eq. G)

Where θ_k_ represents the fixed effect of covariate k, TBW is the time-varying body weight, BALB is the baseline serum albumin, BMGIT is the baseline time to positivity in the mycobacterial growth incubator tube, and age denotes the patients' age at baseline.

**Supplementary 3 (S3): The final model NONMEM code**

$DATA data.csv

$SUBROUTINE ADVAN13 TOL=6

$MODEL COMP=(DP)

$PK

ETAB3 = (EXP(ETA(3))**THETA(14) - 1) / (THETA(14)) ; BOXCOX AMP

;===================== Covariates ======================================

; Base MGIT

MGIT = MTTP

IF(MGIT.EQ.-99) MGIT = 230.5 ; Median MTTP

COVMGIT = (MGIT/230.5)**THETA(10)

; AGE

COVAGE = (AGE/33)**THETA(11)

; Time varying Body Weight

DBDWT = TWT

IF(TWT.EQ.-99) DBDWT = 56 ; Median time-varying WT

COVWT = (DBDWT/56)**THETA(12)

; Serum albumin

ALBB = ALB

IF(ALB.EQ.-99) ALBB = 35 ; median Albumin

COVALB = (ALBB/35)**THETA(13)

; STUDY on HR

COVSTUDY = 0 ; STUDY 208

IF(STUDYNM.EQ.209) COVSTUDY = THETA(15)

;===================== Drug effect =====================================

; M2 effect

M2CONC = CONCM2

IF(M2CONC.EQ.-99) M2CONC = 0

EMAXM2 = THETA(8)

EC50M2 = THETA(9) * EXP(ETA(4))

M2EF = EMAXM2*M2CONC/(EC50M2+M2CONC)

;===================== Heart rate (HR) =================================

TVBASEHR = THETA(1) ; Baseline HR

BASEHR = TVBASEHR * EXP(ETA(1)) * (COVMGIT)

TVSSHR = THETA(2) ; Steady state HR

SSHR = TVSSHR * EXP(ETA(2)) * (COVAGE)

;===================== Disease progression =============================

TVTPROG = THETA(3) ; Half time to steady state HR

TPROG = TVTPROG

KPROG = LOG(2)/TPROG

;===================== Diurnal rhythm ===================================

PI = 22/7

AMP24 = THETA(4) * EXP(ETAB3) ; AMPLITUDE OF COS-RYTHM PERIOD 24 HR

PEAKT24 = THETA(5) ; PEAK OF COS-RYTHM PERIOD 24 HR

AMP12 = THETA(6) * EXP(ETAB3) ; AMPLITUDE OF COS-RYTHM PERIOD 12 HR

PEAKT12 = THETA(7) ; PEAK OF COS-RYTHM PERIOD 12 HR

DIUR24 = AMP24*COS((CTIME-PEAKT24)*2*PI/24) ; COSINE PERIOD 24 HR

DIUR12 = AMP12*COS((CTIME-PEAKT12)*2*PI/12) ; COSINE PERIOD 12 HR

DIUR = DIUR24 + DIUR12

;===================== Initial Compartment =============================

A_0(1) = BASEHR

$DES

DADT(1) = KPROG*SSHR - KPROG*A(1)

$ERROR

HR = (A(1) + DIUR) * (1-M2EF) * (COVWT) * (COVALB) * (1+COVSTUDY)

IPRED = HR

IRES= DV-IPRED

SD = SQRT(SIGMA(1,1)*IPRED**2)

IF(SD.EQ.0) SD = 1

IWRES=IRES/SD

; Time conversion for vpc

WEEK = TIME/24/7

IF(REPL.EQ.1) Y = IPRED*(1+EPS(1)*EXP(ETA(5))) + EPS(2)*EXP(ETA(6))

IF(REPL.EQ.2) Y = IPRED*(1+EPS(1)*EXP(ETA(5))) + EPS(3)*EXP(ETA(6))

IF(REPL.EQ.3) Y = IPRED*(1+EPS(1)*EXP(ETA(5))) + EPS(4)*EXP(ETA(6))

$THETA (0,81.9329399915863) ; 1 TVBASEHR

$THETA (0,72.4505281344159) ; 2 TVSSHR

$THETA (0,1245.98410968949) ; 3 TVTPROG

$THETA (0,6.47421428549114) ; 4 TVAMP24

$THETA (0,15.7918504320819,24) ; 5 PEAKTIME24

$THETA (0,1.63298899090787) ; 6 TVAMP12

$THETA (0,9.70153789952506,12) ; 7 PEAKTIME12

$THETA (0,0.186853211451022,1) ; 8 EMAX M2

$THETA (0,2528.92458608684) ; 9 EC50 M2

$THETA -0.0449214764775003 ; 10 COV MGIT

$THETA 0.0803140206987366 ; 12 COV AGE

$THETA -0.20223945169393 ; 14 COV DWT

$THETA -0.210849539542874 ; 15 COV ALB

$THETA -0.756532011901003 ; 16 BOXCOX AMP

$THETA (-1,0.0458796767700633) ; 17 COV209

$OMEGA BLOCK(3)

0.0229138413793555 ; OM 1 BASEHR

0.00880144006328663 0.0234660101787918 ; OM 2 SSHR

-0.0330181644192775 -0.0527972479677611 0.622789447933392 ; OM 3 AMP

$OMEGA 4.56700327857422 ; OM 4 EC50 M2

$OMEGA BLOCK(2)

0.0532970947026231 ; OM 5 RUV

0.0327081564734351 0.144412004541213 ; OM 6 REPL RUV

$SIGMA 0.00710846357258173 ; SIG PROP

$SIGMA BLOCK(1)

7.46031640740732 ; SIG ADD REPL

$SIGMA BLOCK(1) SAME

$SIGMA BLOCK(1) SAME

$ESTIMATION MAXEVAL=9999 PRINT=1 SIGL=6 NSIG=2 NOABORT METHOD=1

INTERACTION

$COVARIANCE UNCONDITIONAL

**Supplementary 4 (S4): Parameter estimates from the final HR model without the M2 Effect**

| **Description** | | **Parameter Estimate (%RSE) [95%CI]** | **IIV %CV (%RSE)** |
| --- | --- | --- | --- |
| Time on treatment | Baseline HR (bpm) | 78 (1.2) | 15.2 (4) |
|  | Recovered HR (bpm) | 72.1 (1.4) | 15.2 (5) |
|  | T_prog_ (weeks) | 5.88 (17.2) |  |
| Circadian rhythm | Amplitude 24 h (bpm) | 6.2 (12.9) | 93.1 (9) |
|  | Peak time 24 h (clock time) | 15.7 (1.3) |  |
|  | Amplitude 12 h (bpm) | 1.68 (18.5) | 93.1 (9) |
|  | Peak time 12 h (clock time) | 10.2 (4.5) |  |
|  | Box-cox shape for IIV amplitudes | −0.79 (22.4) |  |
| Covariate effects | Effect of study on HR (Study C209 vs C208) | 0.04 (37.1) |  |
|  | Effect of time-varying body weight on HR | −0.19 (15.6) |  |
|  | Effect of baseline serum albumin on HR | −0.22 (17.4) |  |
|  | Effect of baseline TTP MGIT on baseline HR | −0.04 (23.8) |  |
|  | Effect of age on recovered HR | 0.07 (35.1) |  |
| Residual error model | Proportional RUV (%) | 8.4 (2) | 23.6 (7) [15.5] |
|  | Additive replicated-specific RUV (bpm) | 2.7 (1.9) | 38.3 (4) [7] |

bpm = beats per minutes, CI = confidence interval, CV = coefficient of variation, h = hour, HR = heart rate, IIV = inter-individual variability, TTP = time to positivity, MGIT=mycobacteria growth indicator tube, RSE=residual standard error; RUV=residual unexplained variability, T_prog_=time to reach 50% of recovered HR, %CV = $\sqrt{e^{\omega^{2}}-1}$

**Supplementary 5 (S5): Parameter estimates of the final HR model for validation datasets**

|  | **Parameter Estimate (%RSE) [95%CI]** | |
| --- | --- | --- |
| **Description** | **A5343** | **PROBeX** |
| Baseline HR (bpm) | 83 (1%) [80.7, 85.4] | 85.4 (2%) [82.9, 88] |
| Recovered HR (bpm) | 63.3 (2%) [60.2, 66.4] | 73.1 (3%) [68.8, 77.4] |
| T_prog_ (weeks) | 8.51 (14%) [6.15, 10.88] | 9.46 (17%) [6.34, 12.6] |
| Proportional RUV (%) | 9.7 (7%) | 9.7 (6%) |
| Additive replicated-specific RUV (bpm) | 3 (7%) | 2.5 (5%) |

HR=heart rate, T_prog_=time to reach 50% of recovered HR, bpm = beats per minutes, CI = confidence interval, RSE=residual standard error; RUV=residual unexplained variability

**Supplementary 6 (S6): Relationship of QTc vs HR Stratified by Time Visit from C208 & C209 datasets**


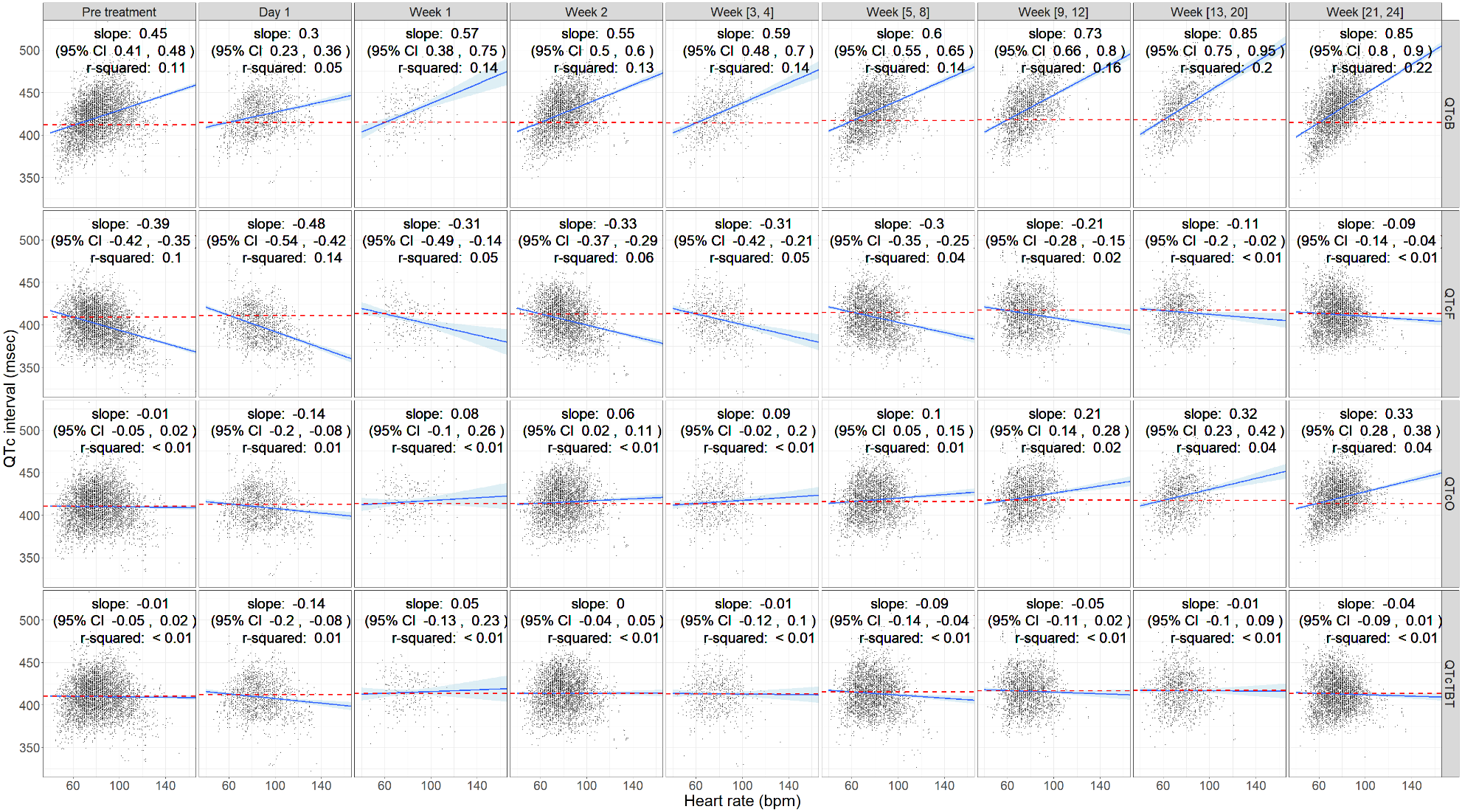


Where the linear regression line is shown in blue, with the 95% confidence interval represented by the light blue shaded area, the red dashed line indicates the QTc at a heart rate of 60 bpm. CI: confidence interval, HR: heart rate, QTcB: QT correction with Bazett’s formula (0.5), QTcF: QT correction with Fridericia’s formula (0.33), QTcO: QT correction with Olliaro’s formula (0.4081), QTcTBT QT correction with a time-varying correction formula, Tprog: time to reach 50% of recovered HR. The end of treatment: week 24.

**Relationship of QTc vs HR Stratified by Time visit from A5343**

**
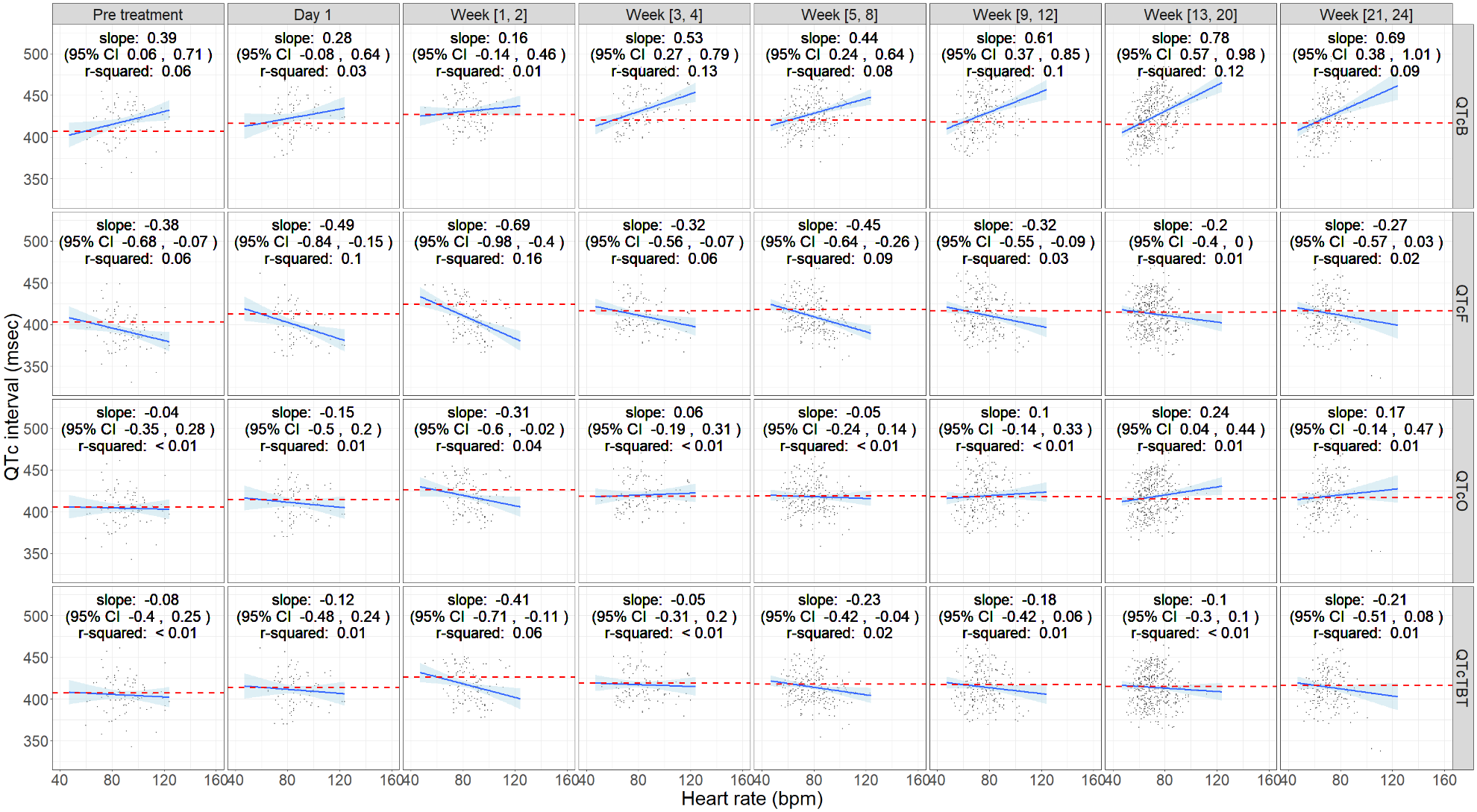
**

Where the linear regression line is shown in blue, with the 95% confidence interval represented by the light blue shaded area, the red dashed line indicates the QTc at a heart rate of 60 bpm. CI: confidence interval, HR: heart rate, QTcB: QT correction with Bazett’s formula (0.5), QTcF: QT correction with Fridericia’s formula (0.33), QTcO: QT correction with Olliaro’s formula (0.4081), QTcTBT QT correction with a time-varying correction formula, Tprog: time to reach 50% of recovered HR. The end of treatment: week 24.

**Relationship of QTc vs HR Stratified by Time visit from PROBeX**

**
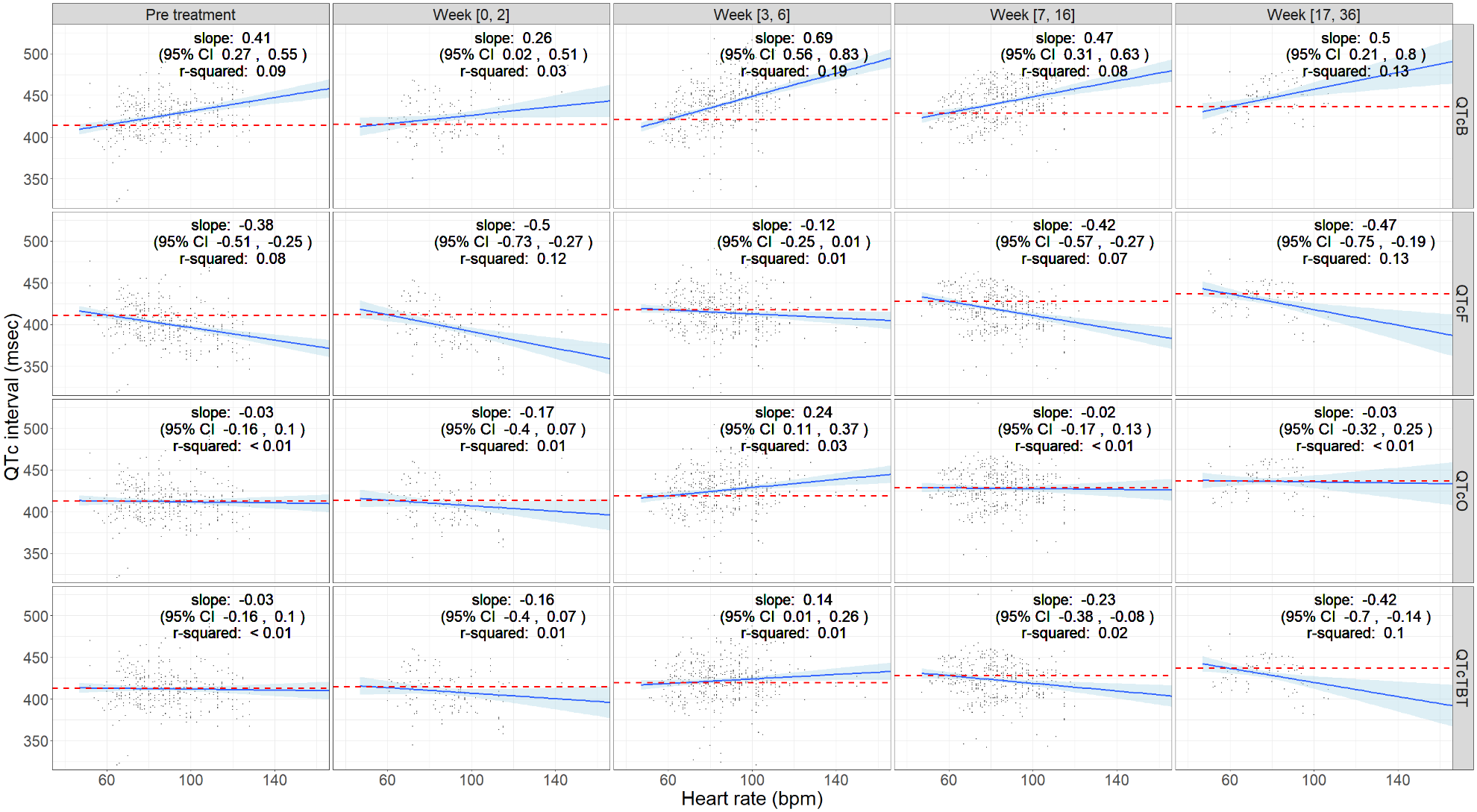
**

Where the linear regression line is shown in blue, with the 95% confidence interval represented by the light blue shaded area, the red dashed line indicates the QTc at a heart rate of 60 bpm. CI: confidence interval, HR: heart rate, QTcB: QT correction with Bazett’s formula (0.5), QTcF: QT correction with Fridericia’s formula (0.33), QTcO: QT correction with Olliaro’s formula (0.4081), QTcTBT QT correction with a time-varying correction formula, Tprog: time to reach 50% of recovered HR. The end of treatment: week 24.

**Supplementary 7 (S7): Relationship of QTc vs HR Stratified by Time Visit for off-treatment (placebo) from C208 datasets**

**
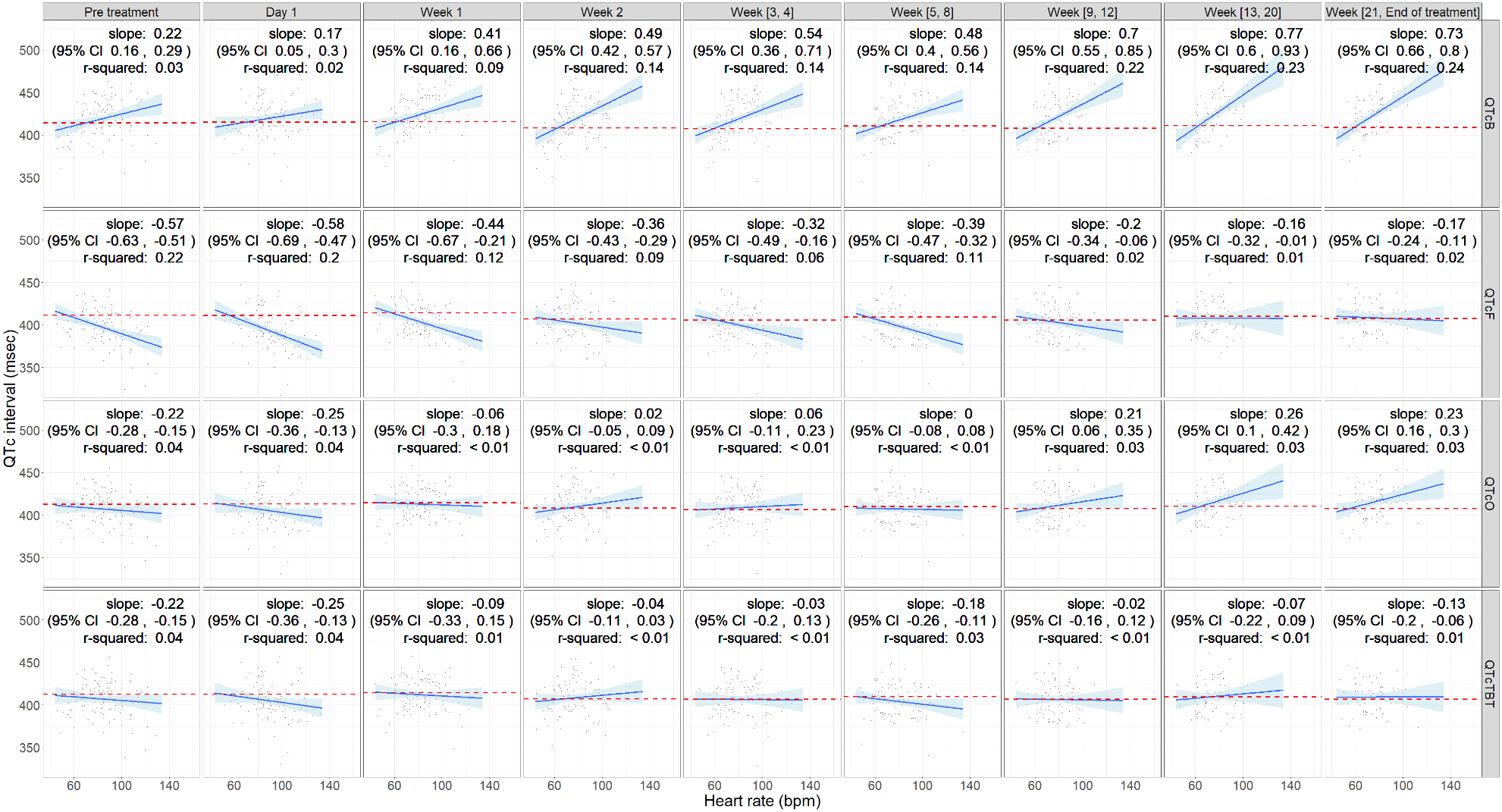
**

Where the linear regression line is shown in blue, with the 95% confidence interval represented by the light blue shaded area, the red dashed line indicates the QTc at a heart rate of 60 bpm. CI: confidence interval, HR: heart rate, QTcB: QT correction with Bazett’s formula (0.5), QTcF: QT correction with Fridericia’s formula (0.33), QTcO: QT correction with Olliaro’s formula (0.4081), QTcTBT QT correction with a time-varying correction formula, Tprog: time to reach 50% of recovered HR. The end of treatment: week 24.

**Supplementary 8 (S8): Relationship of QTc vs HR Stratified by Time visit for a time-varying correction method calculated with the typical, upper and lower boundary of 95%CI of T_prog_ from the pooled development and validation datasets**


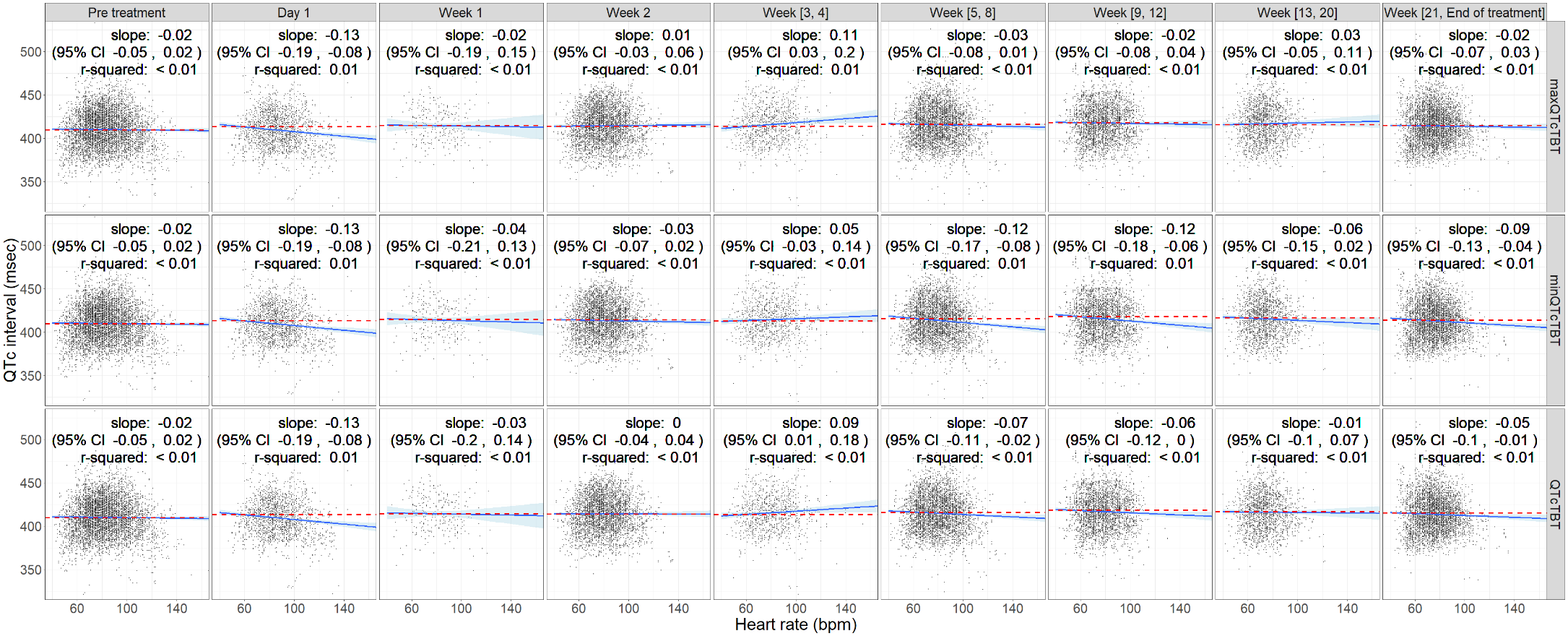


Where the linear regression line is shown in blue, with the 95% confidence interval represented by the light blue shaded area, the red dashed line indicates the QTc at a heart rate of 60 bpm. CI: confidence interval, HR: heart rate, QTcTBT: time-varying-corrected QT, T_prog_: time to reach 50% of recovered HR. maxQTcTBT: an upper boundary of estimated T_prog_, 95% Cl, 10.27 weeks to correct QT interval, the minQTcTBT: a lower boundary of estimated T_prog_, 95% Cl, 5.17 weeks to correct QT interval, the QTcTBT: a typical estimated T_prog_, 7.74 weeks to correct QT interval, and the end of treatment: week 24.

**Supplementary 9 (S9): Summary Statistics for absolute change from baseline(Δ), placebo-corrected change from baseline (ΔΔ) of QTcF and QTcTBT at Different Time Intervals**

| **Times interval** | **Mean (SD)** | | | |
| --- | --- | --- | --- | --- |
|  | **Placebo**  **(C208)** | | **Bedaquiline**  **(C208+C209)** | |
|  | **QTcF** | **QTcTBT** | **QTcF** | **QTcTBT** |
| Pre-treatment | 397.5 (18.6) | 407.9 (17) | 402.7 (18.6) | 411.7 (18.8) |
| Week [0, 1] | 397.7 (18.1) | 408.1 (17) | 402.5 (19) | 412.7 (18.8) |
| Week 2 | 400 (16.5) | 408.1 (16.1) | 410.9 (17.6) | 417.9 (17.3) |
| Week [3, 4] | 399.4 (18.5) | 405.6 (18) | 411.1 (19.7) | 416.4 (19.6) |
| Week [5, 8] | 402 (15.2) | 406.5 (14.7) | 413.5 (18.4) | 417.6 (18.4) |
| Week [9, 12] | 405.1 (17.4) | 407.9 (17.6) | 416 (19.2) | 418.7 (19.2) |
| Week [13, 20] | 407.4 (17.4) | 408.9 (17.4) | 415.5 (19.7) | 417.1 (19.6) |
| Week [21,24] | 406.1 (15.8) | 407.1 (15.7) | 416.9 (18.5) | 417.8 (18.5) |
|  | **ΔQTcF** | **ΔQTcTBT** | **ΔQTcF** | **ΔQTcTBT** |
| Pre-treatment | **-** | **-** | **-** | **-** |
| Week [0, 1] | 0.1 (12.4) | 0.2 (11.6) | 0.1 (10.8) | 1.4 (10.6) |
| Week 2 | 2.4 (14.9) | 0.2 (13.6) | 8.5 (12.3) | 6.7 (11.9) |
| Week [3, 4] | 2.1 (12.2) | -2 (11.9) | 8.8 (14.6) | 5 (14.7) |
| Week [5, 8] | 4.7 (13.9) | -1.1 (12.9) | 10.8 (15.1) | 5.9 (14.8) |
| Week [9, 12] | 5.1 (16.1) | -2.4 (14.7) | 12 (14.7) | 5.8 (14.4) |
| Week [13, 20] | 7.3 (15) | -1.3 (13.7) | 12.1 (17.6) | 4.7 (17.5) |
| Week [21,24] | 7.4 (12.6) | -1.8 (12) | 13.2 (15.9) | 5.3 (15.8) |
|  | **C208** | | | |
|  | **ΔΔQTcF** | **ΔΔQTcTBT** | **ΔΔQTcF** | **ΔΔQTcTBT** |
| Pre-treatment | **-** | **-** | **-** | **-** |
| Week [0, 1] | - | - | 1 (11.8) | 1.1 (11.3) |
| Week 2 | - | - | 5.7 (13.7) | 5.5 (12.2) |
| Week [3, 4] | - | - | 5.2 (15) | 5.5 (14.1) |
| Week [5, 8] | - | - | 7 (13.2) | 7.6 (11.9) |
| Week [9, 12] | - | - | 9.2 (16.2) | 9.3 (15.5) |
| Week [13, 20] | - | - | 7.1 (14.3) | 7.2 (13.8) |
| Week [21,24] | - | - | 5.8 (12.7) | 6 (12.6) |

**Supplementary 10 (S10):** **Example of QTcTBT calculation**

| Weeks after treatment start | Time-varying Correction factor | Example cases | | | |
| --- | --- | --- | --- | --- | --- |
|  |  | QT = 400, HR = 80 | | QT = 450, HR = 100 | |
|  |  | QTcF | QTcTBT | QTcF | QTcTBT |
| 0 | 0.4081 | 440 | 450 | 533 | 554 |
| 1 | 0.4014 |  | 449 |  | 552 |
| 2 | 0.3953 |  | 448 |  | 551 |
| 3 | 0.3897 |  | 447 |  | 549 |
| 4 | 0.3846 |  | 447 |  | 548 |
| 5 | 0.3799 |  | 446 |  | 546 |
| 6 | 0.3756 |  | 446 |  | 545 |
| 7 | 0.3717 |  | 445 |  | 544 |
| 8 | 0.3682 |  | 445 |  | 543 |
| 9 | 0.3649 |  | 444 |  | 542 |
| 10 | 0.3619 |  | 444 |  | 541 |
| 11 | 0.3592 |  | 444 |  | 541 |
| 12 | 0.3567 |  | 443 |  | 540 |
| 13 | 0.3544 |  | 443 |  | 539 |
| 14 | 0.3523 |  | 443 |  | 539 |
| 15 | 0.3504 |  | 442 |  | 538 |
| 16 | 0.3486 |  | 442 |  | 538 |
| 17 | 0.347 |  | 442 |  | 537 |
| 18 | 0.3456 |  | 442 |  | 537 |
| 19 | 0.3442 |  | 442 |  | 537 |
| 20 | 0.343 |  | 441 |  | 536 |
| 21 | 0.3419 |  | 441 |  | 536 |
| 22 | 0.3409 |  | 441 |  | 536 |
| 23 | 0.34 |  | 441 |  | 535 |
| 24 | 0.3391 |  | 441 |  | 535 |
| 25 | 0.3383 |  | 441 |  | 535 |
| 26 | 0.3376 |  | 441 |  | 535 |
| 27 | 0.337 |  | 441 |  | 535 |
| 28 | 0.3364 |  | 441 |  | 534 |
| 29 | 0.3358 |  | 441 |  | 534 |
| 30 | 0.3353 |  | 441 |  | 534 |
| 31 | 0.3349 |  | 440 |  | 534 |
| 32 | 0.3344 |  | 440 |  | 534 |
| 33 | 0.3341 |  | 440 |  | 534 |
| 34 | 0.3337 |  | 440 |  | 534 |
| 35 | 0.3334 |  | 440 |  | 534 |
| 36 | 0.3331 |  | 440 |  | 533 |

Where CF = $0.4081-0.0781(1-e^{\frac{-ln2 \times t}{7.74}})$ , QTcTBT = QT_uncorrected_/RR^CF(t)^, QTcF = QT_uncorrected_/RR^0.33^
